# Automatic background animation generation aligned with LLM-generated lyrics for children’s songs

**DOI:** 10.1038/s41598-025-30139-6

**Published:** 2025-12-27

**Authors:** Sanghyuck Lee, Timur Khairulov, Ye-Chan Park, Wangduk Seo, Jaesung Lee

**Affiliations:** 1 Department of Artificial Intelligence, Chung-Ang University, Seoul, Republic of Korea; 2 Division of AI Computer Science and Engineering, Kyonggi University, Suwon, Republic of Korea; 3 AI/ML Innovation Research Center, Chung-Ang University, Seoul, Republic of Korea

**Keywords:** Engineering, Mathematics and computing

## Abstract

Media content creation is a labor-intensive and expensive process requiring significant time. Recent developments in artificial intelligence have introduced generative models, which have significant potential in the entertainment industry. Meanwhile, demand for video content tailored to children’s songs has steadily increased, reflecting their significant contribution to early education and entertainment. In this paper, we present a generative model-based approach to automated video creation for children’s songs. The proposed pipeline consists of three key steps: generating lyrics using a language model, producing background images with a diffusion model, and overlaying dynamic visual effects to enhance the final output. Our experiments include a comparison of conventional diffusion models and prompt engineering methods, highlighting the superior performance of CascadeSD and the efficacy of landscape or image-style prompting. Lastly, we provide experimental results comparing text-to-video models with our pipeline. The code for our project is available in the following repository: https://github.com/KhrTim/BAGen.

## Introduction

Children’s songs have long been used as an effective medium for nurturing children’s future behavior, social integration, and emotional development^1^. Throughout the history of human society, early childhood education has been a significant factor influencing the personality formation of children. Specifically, children’s songs are both educational and entertaining, forming a traditional method for supporting early learning^2^. With societal evolution, lifestyle changes, and technological advancements, conventional educational methods have adapted to modern conditions by incorporating media technologies into early childhood education^3^. In addition, the rapid development of generative models has elevated the entertainment industry, creating new opportunities for innovative educational and entertainment content^4^.

Media content creation has traditionally relied on the collaborative efforts of multidisciplinary teams, each contributing unique expertise to the process^5^. Furthermore, this process typically requires significant time and money^6^. These constraints and requirements push businesses to seek and adopt novel methods that preserve the quality of work while reducing the cost and creation time. Hence, there is a growing demand for practical generation tools that can automate and enhance the workflows of media creation teams^7^. Furthermore, recent advancements in generative models show their ability to produce high-quality, visually appealing images^8^. Generative models are revolutionizing digital media production by streamlining routine tasks, enabling creators to focus on the more creative and essential aspects of media creation. Such changes are also noticeable in content creation companies targeting children audiences^9^.

Creating background animations for children’s songs is a complex process, which involves interpretingll architecture of the proposed method. unique linguistic and emotional elements. Translating these elements into effective prompts for generative models is particularly challenging^10^. Moreover, existing text-to-video and animation systems are primarily designed for general-purpose media and often lack the controllability, semantic alignment, and safety constraints required for educational or child-oriented content^11,12^. They typically operate as monolithic black-box models that map text directly to video, making it difficult to adapt them for domain-specific storytelling, mood alignment, or age-appropriate visuals^13^. To address these challenges, we propose a modular and extensible pipeline–BAGen–that explicitly focuses on aligning visual generation with lyrical and emotional cues in children’s songs. Unlike existing end-to-end text-to-video systems, BAGen decomposes the task into interpretable components–lyrical understanding using LLMs, model benchmarking, and dynamic effect integration–enabling controllable and expressive media generation that aligns with pedagogical objectives for children’s content.

The contributions of this study can be summarized as follows.

- We propose BAGen, a modular pipeline for generating background animations of children’s songs, integrating LLM-based lyric interpretation with diffusion-driven visual synthesis tailored to child-appropriate themes.
- We design a prompt-engineering framework that aligns lyrics and visuals, ensuring semantic coherence and safety while avoiding textual artifacts and culturally sensitive elements.
- We conduct a systematic comparison of five diffusion models under identical conditions, identifying CascadeSD as the most effective for imaginative, lyric-driven image generation.
- We develop a semi-automated animation workflow that overlays dynamic motion effects on generated backgrounds, producing coherent looping visuals with human-in-the-loop control for quality assurance.

To demonstrate the effectiveness of our pipeline, we provide quantitative measures for comparing our pipeline output with text-to-video models. In addition, we provide a quantitative evaluation of images generated for children’s song visualization. Focusing on children’s songs, we sought to improve the model’s performance by creating visually engaging and thematically relevant content.

## Related work

Text-to-image (T2I) generation is a key area in generative modeling, aiming to produce images that match textual prompts. These models are widely used in content and art creation, saving time and enabling non-designers to create visual prototypes without relying heavily on design teams^14^.

Early T2I approaches were based on generative adversarial networks (GANs)^15^, including models like DM-GAN^16^, Attn-GAN^17^, and Stack-GAN^18^, which introduced multi-stage pipelines for high-resolution image synthesis. GANs consist of a generator and a discriminator that train adversarially, with text inputs converted into latent vectors considered by the discriminator.

With the rise of transformers, models like iGPT^19^ and DALL-E^20^ extended transformer architectures to image synthesis. DALL-E uses a two-stage pipeline: a variational autoencoder compresses images into latent space, and text embeddings are combined with these to generate images progressively. Semantic alignment between text and image is assessed using CLIP^21^, which bridges visual and textual modalities. Advances in guidance mechanisms and multimodal datasets have since accelerated the development of diffusion-based models^22^.

With the introduction of parameterized Markov chains into the process, a new generation of models has emerged: denoising diffusion probabilistic models (DDPM)^23^. Diffusion models outperform traditional GAN approaches in terms of both the quality and the amount of detail in the generated images. The first remarkable milestone model that applied the DDPM to T2I tasks was GLIDE^24^. CLIP^21^ guidance and classifier-free guidance (CFG) mechanisms were explored in that study, and the results showed that CFG provides better results in generating realistic and detailed images. Adopting CFG, Imagegen^25^ uses a pre-trained large language model (LLM) as a text encoder instead of training task-specified text encoders, as in GLIDE^24^. This study suggests that developing LLMs also increases the quality of the generated scenes and their correspondence with text prompts. Latent diffusion models (LDMs)^26^ have emerged as solutions for producing high-quality results in environments with limited computational resources. This technique applies a denoising process to the latent space of pre-trained autoencoders. The application of the LDM with a powerful pre-trained auto-encoder was demonstrated for stable diffusion.

Despite the effectiveness and widespread applicability of T2I models in marketing^27^, digital art^28^, and the gaming industry^29^, these models still lack the ability to capture dynamic content. This gap is being addressed by recent developments in text-to-video (T2V) models.

Recent research on text-to-video generative methods falls into two major groups: end-to-end and two-stage models. In end-to-end text-to-video generation^30^, a single model performs all necessary steps to produce the visual output from the textual prompt. One recent model that falls into this category is CogVideoX^31^, which introduces several novel design decisions, such as a 3D Variational Autoencoder, an expert transformer, and multi-resolution frame packing. Another end-to-end model is presented by Jin Y. et al.^32^, who address the high resource usage of generative models by introducing a unified pyramidal flow matching algorithm. Ang W. et al.^33^ introduced WAN, which features a novel spatio-temporal variational autoencoder, scalable pre-training strategies, and large-scale data curation to achieve remarkable performance.

Two-stage methods first generate an image from a text prompt^34^, then use it to produce a video sequence^35^. While recent T2V models^30,36^ have gained attention, they face challenges in generating background animations for children’s songs^37,38^. Key issues include poor handling of children’s lyrics and the inclusion of irrelevant elements like text visualizations in the output.

Despite the development of various task-specific methods, there remains a notable lack of research focused on generating background animation for children’s songs, highlighting an underexplored area in generative modeling.

## Method

**Figure 1:**
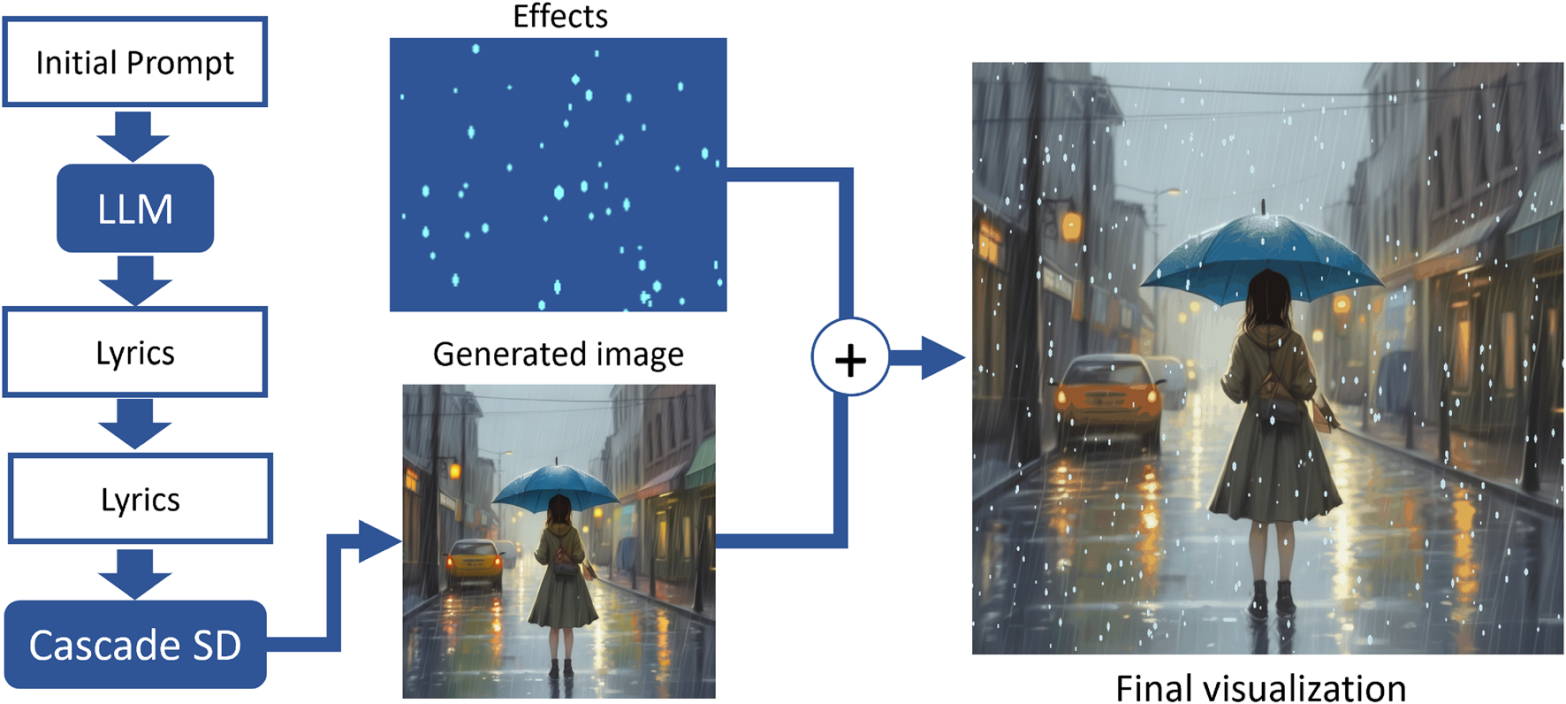
Overall architecture of the proposed method. The generated images are produced by CascadeSD.

This section describes the proposed pipeline for generating background animation of children’s songs in detail. Our pipeline consists of three stages, each dedicated to a specific task: generating lyrics using a language model, generating background images with cascade diffusion and prompt engineering, and applying overlay effects. Figure 1 shows a high-level overview of the sequence of steps included in a pipeline and their dependencies.

### Lyrics generation

The first step of our pipeline is the generation of lyrics using large language model (LLM). Various general-purpose LLMs are available due to the continuous interest in natural language processing applications. Open-source models such as Pathways Language Model^39^, Bidirectional Encoder Representations from Transformer^40^, and Large Language Model Meta AI^41^ offer extensive customization potential and were trained on broad and general-purpose corpora. These models are representative LLMs designed for general language understanding and generation tasks.

### Cascade diffusion

After lyric generation is completed and a single or set of prompts is compiled, a generative model produces a background image matching the context and special conditions in the prompt. High-performance T2I models can generate high-quality images but lack the constraints to produce image styles suitable for children’s songs. Hence, prompt engineering is essential in the current stage because textual descriptions of the scene or characters in the created lyrics directly affect the generated image quality and completeness of the final scene. Specifically, by incorporating specific art styles or keywords into the lyrics, we can enhance the background image generative model to produce outputs well-suited for a background animation of children’s songs. The experimental section will cover this in detail, and here, we discuss the diffusion model, which is primarily used for generating background images.

Diffusion models incrementally convert input text, such as song lyrics, into corresponding visual representations. These models operate by progressively rearranging random noise into meaningful images guided by a textual input. Through iterative processes, they bridge the semantic gap between language and visuals, effectively generating images reflecting themes, emotions, or details conveyed in the input text. The following explains the T2I process based on DDPM, one of the widely used formulations of diffusion models. The main approach underlying the DDPM is defined by a forward process^42^, in which the data $x_0 \sim q(x_0)$ are gradually destroyed in $T$ steps

$$\begin{aligned} q(x_{1:T}|x_0)=\prod _{t=1}^{T}N(x_t; \sqrt{1-\beta _{t}}x_{t-1},\beta _{t}I), \end{aligned}$$

and the parametrized reverse process that restores the image

$$\begin{aligned} p_\theta (x_0)=\int p(x_T)\prod _{t=1}^{T} N(x_{t-1};\mu _\theta (x_t, t),\sum _\theta (x_t,t)) d x_{1:T}. \end{aligned}$$

By optimizing the evidence lower bound - $L_\theta (x_0) \le log p_\theta (x_0)$, the reverse process can be trained to approximate the joint distribution created by the forward process. This can be written as

$$\begin{aligned} L_\theta (x_0)=\mathbb {E}_q\left[ L_T(x_0)+\sum _{t>1}D_{KL}(q(x_{t-1}|x_t,x_0) || p_\theta (x_{t-1}|x_t))-logp_\theta (x_0|x_1)\right] . \end{aligned}$$

For T2I generation, the model learns the conditional distribution $p_\theta (x_0|c)$ to include Condition *c* as an additional parameter for the reverse process^43^ through

$$\begin{aligned} p_\theta (x_0|{\textbf {c}})=\int p(x_T)\prod _{t=1}^{T} N(x_{t-1};\mu _\theta (x_t, t,{\textbf {c}}),\sum _\theta (x_t, t, {\textbf {c}})) dx_{1:T}, \end{aligned}$$

and

$$\begin{aligned} L_\theta (x_0|{\textbf {c}})=\mathbb {E}_q\left[ L_T(x_0)+\sum _{t>1}D_{KL}(q(x_{t-1}|x_t,x_0) || p_\theta (x_{t-1}|x_t,{\textbf {c}}))-log p_\theta (x_0|x_1,{\textbf {c}})\right] \end{aligned}$$

Guidance during the image generation process presents a method for high-quality image generation based on a given textual input. In this manner, DDPM generates images by iteratively adding noise that follows a defined distribution; subsequently, an image restoration process is initiated to convert noise into a meaningful image that matches the given prompt.

Several models were considered for inclusion in our pipeline, and through extensive experiments, the CascadeSD^44^ was selected for use in the production environment. CascadeSD introduces an innovative T2I synthesis architecture to enhance efficiency and reduce computational demands. Leveraging a compact semantic image representation to guide the diffusion process significantly lowers GPU usage and accelerates inference. Compared to state-of-the-art models, such as Stable Diffusion 2.1, CascadeSD delivers competitive performance while requiring substantially fewer GPU hours and achieving faster processing times.

### Overlay effects

**Figure 2:**
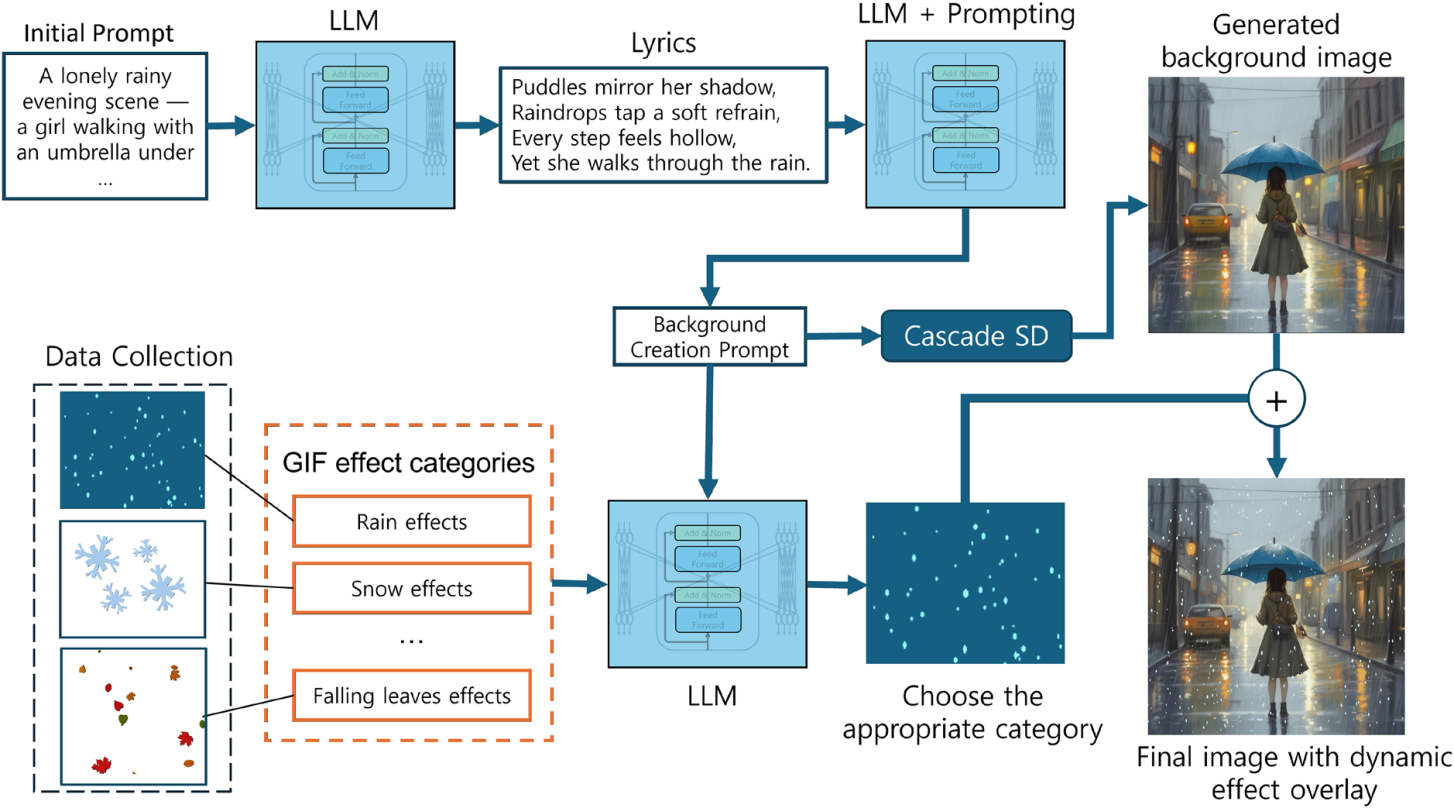
An overview of animated image generation process. The generated background image is produced by CascadeSD.

The last step of our workflow is to add dynamics to the generated scene by applying moving elements supported by image formats with transparency and animation capabilities. This step enhances the visual appeal of generating the aesthetic quality of images, ensuring alignment with lyrical themes and delivering a visually captivating experience. To this end, we gathered visual data featuring effects such as falling leaves, falling flowers, rain, snow, and fireflies. To choose the element that would match the atmosphere of the generated scene, we utilized the LLM. First, we manually grouped the visual effect files into thematic categories, such as rain, snow, and others. Then, the LLM is provided with these category titles along with the prompt that was used to generate a background image. By combining this information into a single input, the LLM could infer and suggest the most appropriate visual effect category for the scene. Once the appropriate category is selected, a specific animation is randomly sampled from the group to introduce variability in the output. Finally, the selected effect is overlaid onto the images generated by CascadeSD. Figure 2 illustrates the animated image-generation method.

This method enables fully automated production of animated images by incorporating the LLM into the effect selection process. By analyzing the same prompt used for background generation, the LLM can infer the context of the scene and select the visual effect category that best aligns with it. The final animation is created by combining the generated background image with the selected effect, resulting in a dynamic and engaging visualization of song lyrics. As a result, the pipeline can produce visually rich and contextually relevant animations that enhance the storytelling and content perception.

## Experiments

We conduct experiments on background image generation for AI-generated lyrics using five state-of-the-art T2I models. The comparison models include CascadeSD^44^, ReCo^45^, TIME^38^, T2I-CompBench^37^, and GlymphControl^46^. The rationale for choosing five compared methods and their drawback is summarized as follows.

- CascadeSD^44^ demonstrates superior quality and efficiency over Stable Diffusion, making it more suitable for creating engaging visuals.
- ReCo^45^ facilitates region-specific control but performs poorly in generating cartoon-style images.
- TIME^38^ mitigates implicit assumptions but produces less aesthetically appealing imaginative scenes.
- T2I-CompBench^37^ improves compositional accuracy but does not align well with children’s visual perception.
- GlyphControl^46^ enhances text readability but may visually overload compositions.

In this study, we did not train or fine-tune any models. All diffusion and LLM-based models were used in their publicly released, pretrained form. Specifically, CascadeSD, GlyphControl, ReCo, TIME, and T2I-CompBench were obtained from their official repositories or Hugging Face model hubs as cited in the corresponding papers. These models were originally trained on large-scale, publicly available image–text datasets such as LAION-5B^47^, COCO^48^, and other open image corpora.

To ensure that the generated outputs were child-safe and culturally appropriate, we employed prompt-engineering techniques to guide both the language and visual generation processes toward content appropriate for young audiences. Prompts were designed to exclude violent, sexual, or culturally sensitive elements and to emphasize friendly, imaginative, and educational themes. In addition, each stage of the pipeline allows human inspection and interaction, providing an additional safeguard through real-time monitoring by the end user. Thus, inappropriate generations can be immediately identified and discarded. No additional dataset collection, filtering, or model retraining was performed.

The evaluation of generated images is not a trivial task because mainly humans can easily identify how well a generated image corresponds to a given prompt and determine the quality of the final scene. However, due to the inconsistency of human judgment and the high labor cost, extensive study is being conducted to find a measure that can emulate human perception of generated scenes. In this study, several evaluation methods were employed to define the best matching model for our task of image generation of children’s song lyrics, including well-known measures such as the CLIP score^49^ and recently released methods, such as CLIP image quality assessment (CLIP-IQA)^50^ and text-to-image faithfulness evaluation with question answering (TIFA)^51^.

**Table 1:** Experimental results for background image generation models.

Model name	CLIP score^49^	CLIP-IQA^50^	TIFA^51^
CascadeSD^44^	**0.347 ± 0.030**	**0.756 ± 0.068**	**0.873 ± 0.203**
GlyphControl^46^	0.318 ± 0.044	0.690 ± 0.092	0.777 ± 0.241
ReCo^45^	0.302 ± 0.045	0.667 ± 0.111	0.771 ± 0.241
T2I-CompBench^37^	0.343 ± 0.031	0.680 ± 0.112	0.832 ± 0.204
TIME^38^	0.332 ± 0.033	0.726 ± 0.101	0.757 ± 0.211

The CLIP score^49^ was used to evaluate the association between an image and a caption by calculating the cosine correlation. This measure demonstrated a strong alignment with human judgment. The CLIP score is defined as

$$\begin{aligned} CLIPScore(I, C) = max(100 * cos(E_I, E_C), 0), \end{aligned}$$

where $E_I$ is a CLIP embedding^21^ for an actual image and $E_C$ is a CLIP embedding^21^ for a given caption. Because the measure output range was inconsistent, the CLIP score results were scaled to fall within a zero-to-one range. Using this approach, the semantic alignment of a given prompt and the corresponding generated image can be numerically measured. Another standard measure used in our evaluation is the CLIP-IQA, which is also based on the CLIP. However, in contrast to the CLIP score, CLIP-IQA^50^ requires only one image for evaluation because this measure was developed to evaluate the quality of the generated scene. CLIP-IQA also works by computing cosine similarity, but in contrast to the CLIP score, it computes the similarity of a given image and a predefined set of semantically opposite pairs of prompts such as ‘Good photo’ – ‘Bad photo’ or ‘Clean photo’ – ‘Noisy photo.’ Thus, the numerical results for the overall image quality can be calculated.

**Figure 3:**
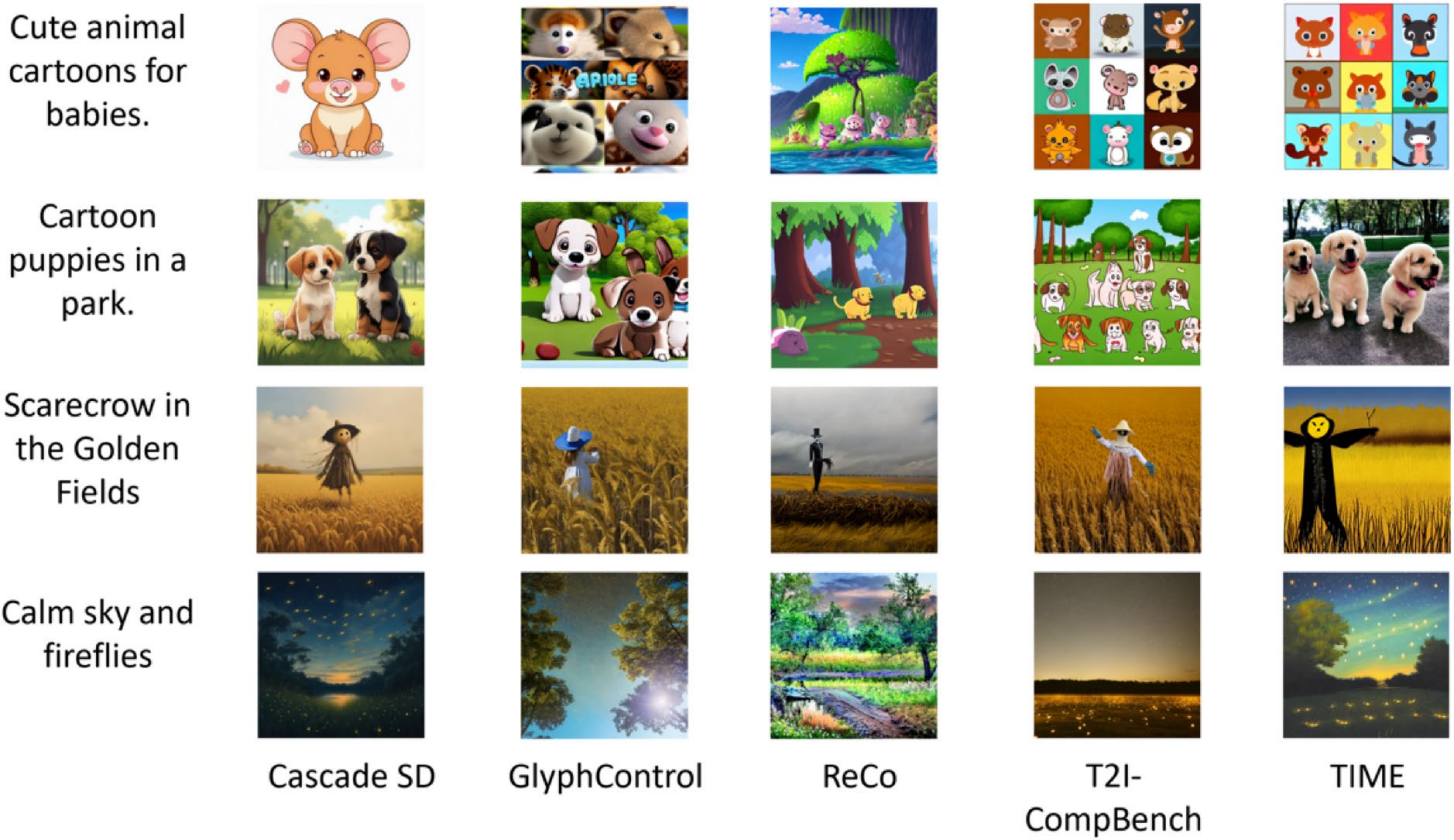
Image generation results of five models using naïve prompts for children song. All images shown are generated by the five models.

**Figure 4:**
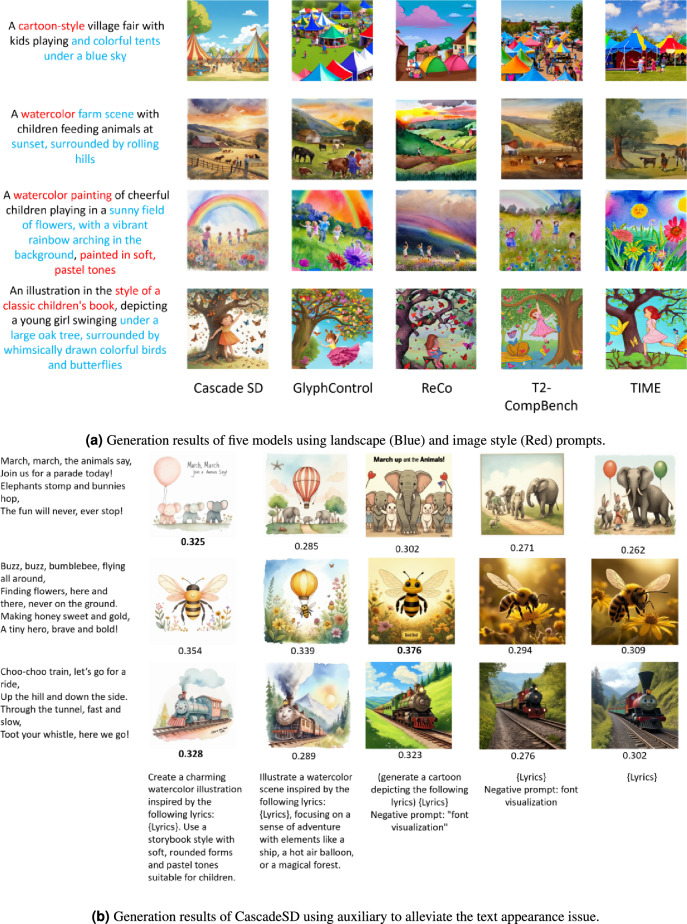
An example of adding keywords related to landscape or image style to the prompt. Each image is generated by the model specified in the corresponding subfigure caption.

**Figure 5:**
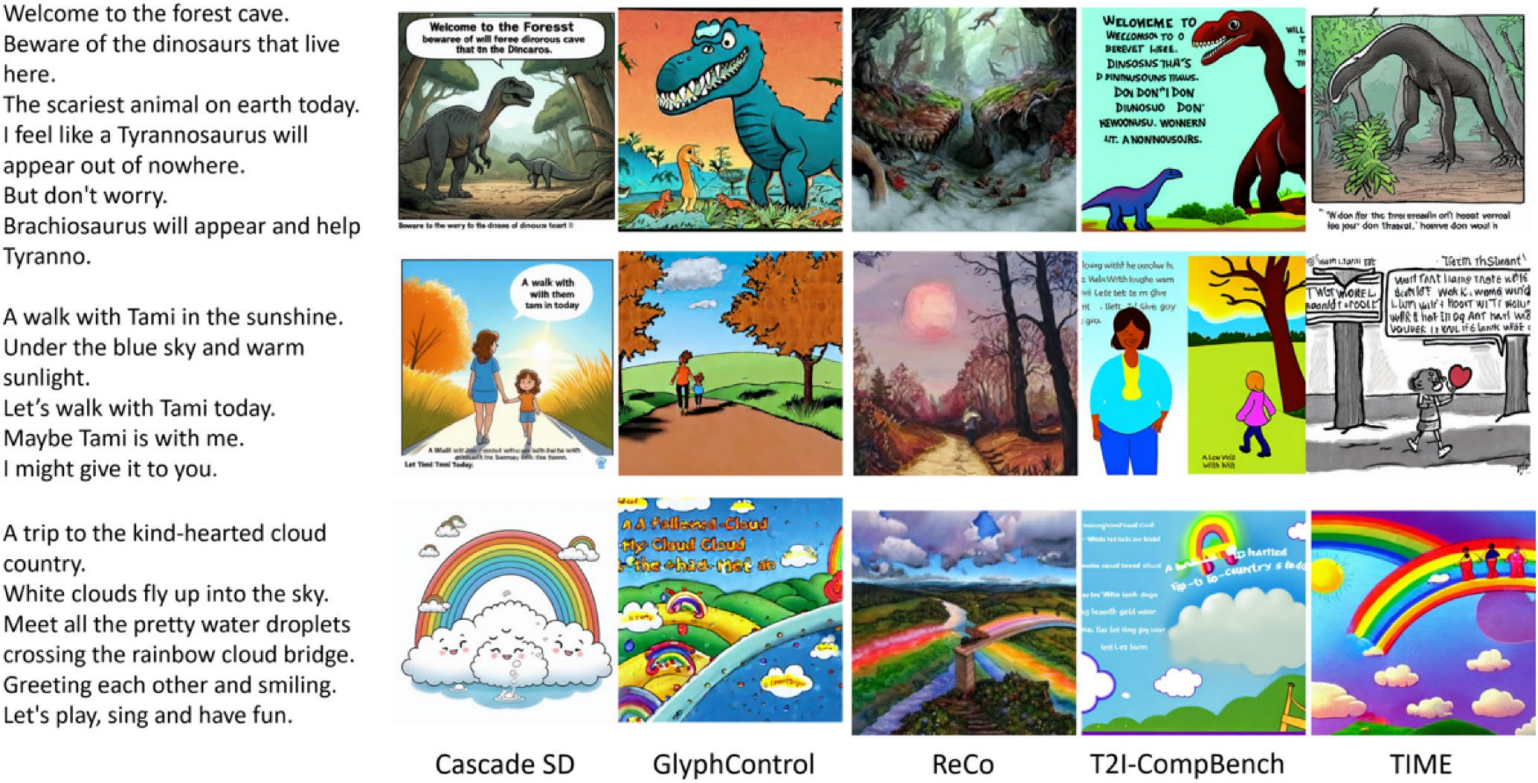
Illustrate a cartoon scene inspired by the following lyrics: $<lyrics>$. All images shown are generated by the five models.

Lastly, our experiments used a novel approach for evaluating the performance of generative models called TIFA. In the first stage, a prompt for image generation is analyzed using the LLM to extract meaningful information about objects, their appearance, scene description, and the overall atmosphere, which is expected in the final image. Then, the LLM generates a list of possible answers to the generated questions. This information is passed on to the second stage of TIFA evaluation, where another model attempts to match the generated image with a set of questions from the first stage. By answering prepared questions via visual question answering and matching answers with the predicted ones, it is possible to evaluate the results of the generative model like human-style evaluation. This two-stage process makes TIFA scores closely correlated with human evaluations, and the results of such an evaluation are easily interpretable for humans. For all the measures used, a higher score indicates a better result.

To ensure reproducibility and fair comparison across models, all stochastic components were controlled using a fixed random seed and deterministic configurations across PyTorch, NumPy, and CUDA environments. Consistent hyperparameter settings were maintained for both lyric generation and image synthesis. For the LLM-based lyric generation (EXAONE-3.5-2.4B-Instruct), text sampling was stabilized by setting a moderate temperature (0.7) and restricting randomness through top-k and top-p parameters. For the diffusion-based image generation (Stable Cascade), identical settings were applied for guidance scale and inference steps across all runs. All experiments were performed with PyTorch 2.5.1 (CUDA 12.4) on an NVIDIA RTX 3090 GPU under deterministic modes. The full configuration details and implementation scripts are included in the Supplementary Materials and publicly available repository.

As demonstrated in Table 1, CascadeSD achieved consistently high scores across all evaluation measures, indicating strong overall performance compared to the other models. In the CLIP Score assessment, CascadeSD obtained a score 0.347 ± 0.030 slightly higher than 0.343 ± 0.031 demonstrated by T2I-CompBench. This result indicates that CascadeSD provides reliable alignment between the input prompt and the generated image, yielding highly relevant visual outputs. Similarly, in the CLIP-IQA, CascadeSD delivered one of the strongest performances, achieving a score of 0.756 ± 0.068. This result indicates that the model is capable of generating high-quality images. TIME followed closely with a respectable score of 0.726 ± 0.101, indicating solid performance, though slightly behind CascadeSD. Lastly, in the TIFA evaluation, CascadeSD (0.873 ± 0.203) and T2I-CompBench (0.832 ± 0.204) were recognized as the top models in terms of prompt-to-image alignment, further validating their strong performance in generating coherent and accurate images based on textual descriptions. A qualitative analysis of the experimental results is presented in depth in the following section, which focuses on prompt engineering.

In addition to the model choice, experiments were conducted to explore the results and limitations of different prompting techniques. During the experiments with different prompting techniques, we identified several limitations associated with certain types of prompts, along with those that produced the desired behavior. Figure 3 presents the results of inputting a naive prompt into text-to-image models (lyrics only), whereas Fig. 4a shows the results after adding keywords related to landscape or image style to the prompt. We observed improved performance by successfully constraining the suit to the children’s songs.

Although models have demonstrated their capability to generate high-quality images aligned with given prompts, a specific issue arises when prompts combine a particular style with children’s song lyrics. The models start by embedding glyphs into the generated images. Such behavior is undesirable and diminishes the overall quality of the scene. These glyphs are typically of low resolution and often contain meaningless text. To address this issue, several experiments were conducted using various prompts and lyrics to identify an effective approach for combining style and lyrics without triggering the inclusion of glyphs. The results are presented in the Fig. 4b. Observations of our experimental results indicate that prompts that would combine style and lyrics without triggering the inclusion of glyphs follow this structure: ‘Illustrate a $<style>$ scene inspired by the following lyrics: $<lyrics>$’ or ‘(generate a cartoon depicting the following lyrics) $<lyrics>$.’ To validate our hypothesis, we generated two sets of images using the following prompts: ‘Illustrate a cartoon scene inspired by the following lyrics: $<lyrics>$’ (Fig. 5) and ‘Illustrate a watercolor scene inspired by the following lyrics: $<lyrics>$’ (Supplementary Fig. S1 online).

Given that the final output of our pipeline is an animated representation of children’s songs, we conducted supplementary experiments using text-to-video models. In this experiment, 28 distinct prompts were generated, each featuring different scene and main actors. These prompts were automatically derived from base phrases such as ‘cat under rain’ or ‘family watching fireworks’ using an LLM, with the goal of producing the most visually compelling descriptions–consistent with the approach employed in our proposed method. Based on our prompt engineering investigations, we curated a set of optimized prompts to ensure high-quality generation. We used seven evaluation measures. Pairwise clip was calculated as an average clip similarity between all pairs of the frames in video model output. Similarly, text alignment was calculated as an average of clip similarities between prompt and each frame of the video. Subject consistency, background consistency, motion smoothness, aesthetic quality and image quality measures are automatically collected with VBench^52^, a benchmark specifically created to evaluate the performance of generative models. In terms of all the measures used, a higher score indicates a better result. For our counterpart methods we selected CogVideoX^31^, Pyramid Flow^32^, WAN^33^ and HotShotXL^53^, the latter of which is explicitly designed to generate GIF animations. Since VRBench requires video format, GIF outputs were converted to video with optimal settings to preserve the original quality.

**Table 2:** Comparison of text-to-video model performance regarding seven evaluation measures.

	Evaluation	BAGen (ours)	CogVideoX	HotShotXL	Pyramid Flow	WAN
	mesaure					
	Pairwise	**0.986 ± 0.015**	0.912 ± 0.054	0.985 ± 0.010	0.964 ± 0.026	0.968 ± 0.027
CLIP	clip					
	Text	**0.313 ± 0.029**	0.244 ± 0.043	0.265 ± 0.03	0.272 ± 0.038	0.253 ± 0.035
	Alignment					
	Subject	0.972 ± 0.029	0.937 ± 0.038	**0.987 ± 0.007**	0.947 ± 0.041	0.946 ± 0.072
	Consistency					
	Background	**0.985 ± 0.013**	0.928 ± 0.041	0.975 ± 0.011	0.968 ± 0.016	0.971 ± 0.025
	Consistency					
VR	Motion	0.992 ± 0.006	0.986 ± 0.011	0.980 ± 0.014	**0.995** ± **0.003**	0.988 ± 0.008
Bench	Smoothness					
	Aesthetic	**0.779 ± 0.073**	0.355 ± 0.113	0.646 ± 0.089	0.679 ± 0.046	0.654 ± 0.068
	Quality					
	Imaging	0.651 ± 0.102	0.551 ± 0.115	0.622 ± 0.185	0.575 ± 0.098	**0.652** ± **0.078**
	Quality					

Our experimental results, presented in Table 2, demonstrate that the proposed model produces competitive outputs. Specifically, BAGen achieved the highest CLIP-based scores, with a Pairwise CLIP similarity of $0.986\pm 0.015$ and Text Alignment of $0.313\pm 0.029$, outperforming all baselines. In the VR Bench evaluation, BAGen also attained top performance in Background Consistency ($0.985\pm 0.013$) and Aesthetic Quality ($0.779\pm 0.073$), while maintaining comparable results in Motion Smoothness ($0.992\pm 0.006$) and Imaging Quality ($0.651\pm 0.102$). These results suggest that BAGen is a viable approach for generating visually appealing and semantically aligned animations for children’s lyrics. In terms of computational efficiency, the method exhibits consistent generation times, with most runs completing within 40–47 seconds and occasional longer runs (up to 65 seconds) for more complex prompts. These results indicate that the approach is computationally feasible and potentially applicable to real-world, small-scale animation or interactive media generation scenarios.

## Discussion

Owing to the effectiveness of our pipeline, several avenues for potential improvement could enhance the quality of the results. Although the CascadeSD model demonstrated remarkable performance in generating high-resolution images with fine details, our experiments highlighted certain limitations in its ability to handle specific prompts. Despite the overall capability of the model, some prompt scenarios appear to challenge its underlying architecture, leading to suboptimal results. The problematic prompts and their corresponding generated images are shown in Supplementary Fig. S2 online, illustrating the areas where the model performance falls inadequately. These results suggest that while the model is highly effective in many cases, certain situations still require refinement, particularly when considering more complex or abstract requests.

The remarks on the compared methods can be summarized as follows. The GlyphControl generates images with readable text, which suits use cases like ads or signs. However, embedding lyrics in children’s song backgrounds can clutter the visuals and reduce engagement, especially for younger viewers who may struggle with reading. Our experiments also showed that when text placement wasn’t specified, the model produced artifacts that reduced image quality. One of the compared methods, ReCo, focuses on region-specific image generation by incorporating positional embeddings into queries. A disadvantage of this model for imaginary scene generation is that ReCo was fine-tuned on real-world scenes. As a result, cartoon-stylized image generation appears rough and consists mainly of basic shapes, which may not be visually appealing to children. Therefore, this model is not well-suited for generating imaginative scenes. The TIME model addresses implicit assumptions in generative models, but this is less relevant for children’s song backgrounds, which favor imaginary settings. Our experiments also show that TIME produces less visually appealing images for children’s lyrics, making it less suitable for this task. The T2I-CompBench model fine-tunes Stable Diffusion by rewarding accurate image generation for compositional prompts, helping it integrate objects and relationships into complex scenes. However, these scores don’t always align with human perception, and strict prompt alignment is less important for visuals aimed at children. Moreover, the model is based on Stable Diffusion, which is now outdated compared to newer models like Stable Cascade. In contrast, CascadeSD adopts a more modern approach based on the Würstchen architecture, which consists of three sequential stages. The model first generates an image with a high compression ratio using a text-conditional LDM in Stage C. Stage B transforms this representation into a less-compressed latent space using a secondary model that enhances the reconstruction process. Lastly, in Stage A, tokens comprising the latent image are decoded into the final output image. Experimental evaluations show that CascadeSD produces higher-quality results than Stable Diffusion while being more computationally efficient. Despite these advantages, certain failure cases persist, as demonstrated in Supplementary Figure S2, particularly under prompts involving abstract or ambiguous concepts^21^. These instances highlight the need for additional fine-tuning and prompt engineering to enhance reliability across diverse scenarios.^54,55^

While existing text-to-video models such as CogVideoX^31^, HotShotXL^53^, WAN^33^, and Pyramid Flow^32^ demonstrate impressive performance, their architectural objectives fundamentally differ from that of BAGen. These large-scale diffusion transformers achieve temporal coherence through frame-level or autoregressive mechanisms, but they require substantial computational resources and are primarily optimized for general-purpose video synthesis. In contrast, BAGen adopts a modular and interpretable design tailored for children’s song visualization. Our pipeline achieves perceptual motion continuity by overlaying animated effects on diffusion-generated static backgrounds rather than explicitly learning temporal dynamics. While this structure offers lightweight operation, controllability, and semantic coherence, it inherently lacks a dedicated temporal modeling mechanism. Future work will address this limitation by integrating temporal-aware diffusion modules or recurrent frame conditioning to achieve smoother and more consistent video dynamics.

Beyond technical performance, the generated images also hold potential educational benefits for young audiences^56^. The visually engaging and imaginative scenes accompanying children’s songs can support language learning, attention, and emotional connection by reinforcing the meaning of lyrics through concrete imagery^57,58^. Such multimodal presentations are known to enhance comprehension and memory retention in early learning contexts^59^. Moreover, the playful and culturally inclusive designs can foster creativity and curiosity^60^. Although our study focuses primarily on technical validation, future work could involve collaborations with educators to evaluate how automatically generated visuals influence engagement, learning outcomes, and age-appropriate content understanding.

Building on these perspectives, several directions could enhance the diversity and dynamism of the generated outputs^61^. Currently, BAGen generates animated backgrounds corresponding to the lyrics, without animating characters or including text in the visuals. Incorporating character animation and fully dynamic scenes is part of future work. In this context, techniques such as Visual Speech Recognition^62^ could be employed to align lip movements with lyrics, improving realism and engagement in animated characters. A promising avenue is integrating audio alignment with lyrics and visual atmosphere to create a more immersive experience^63^. Future work could explore tighter coupling between visual and audio generation modules, allowing rhythm, melody, and mood to directly influence visual dynamics^64,65^. Additionally, incorporating small-scale human evaluations with educators, parents, or child participants could provide valuable insights into perceptual quality, engagement, and educational effectiveness, complementing automated measures^66^. Ethical considerations are also crucial when generating media for children; thus, future research should include systematic assessment of content safety, bias mitigation, and cultural inclusivity to ensure responsible AI deployment in educational and entertainment contexts^67,68^.

## Conclusion

This paper presented a multimedia tool for generating background animations for children’s songs by integrating diffusion models and large language models (LLMs), which have recently shown strong potential in generative applications. Children’s content offers valuable opportunities for both creativity and education, yet AI-driven video generation remains a challenging and evolving area. Our diffusion-based approach demonstrates promising performance in improving visual quality and highlights new possibilities for the creative use of generative models in this domain.

To identify suitable configurations for our purpose, we conducted experiments with different models and parameter settings, examining their ability to generate high-quality and contextually aligned results. We also explored a dynamic overlay technique to enrich the generated visuals with matching effects. Additionally, we performed comparative experiments with text-to-video systems to assess the feasibility of our approach. While the findings are encouraging, further research is needed to enhance consistency, control, and creativity. In future work, we plan to refine our framework and extend it toward more expressive media generation that aligns with pedagogical objectives for children’s content.

## Supplementary Information

Supplementary Information.

## Data Availability

The code for the generative AI pipeline is available in the BAGen repository at https://github.com/KhrTim/BAGen. The experimental results and generated outputs from this study are included in this published article and supplementary file. No external datasets were used in this research.

## References

[1] Karoly, L. A., Greenwood, P. W., Everingham, S. S., Houbé, J. & Kilburn, M. R. Investing in our children: What we know and don’t know about the costs and benefits of early childhood interventions. *Rand Corporation* (1998).

[2] Jiayin, W. & Sondhiratna, T. Teaching children’s songs in early childhood education. *Asia Pac. J. Religions Cultures* **8**, 605–615 (2024).

[3] Paul, C. D., Hansen, S. G., Marelle, C. & Wright, M. Incorporating technology into instruction in early childhood classrooms: A systematic review. *Adv. Neurodev. Disord.* **7**, 380–391 (2023). 10.1007/s41252-023-00316-7 PMC9918405 36816781

[4] Ruiz-Rojas, L. I., Acosta-Vargas, P., De-Moreta-Llovet, J. & Gonzalez-Rodriguez, M. Empowering education with generative artificial intelligence tools: Approach with an instructional design matrix. *Sustainability* **15**, 11524 (2023).

[5] Fleischmann, K. & Daniel, R. J. Increasing authenticity through multidisciplinary collaboration in real-life scenarios in digital media design education. *CoDesign* **6**, 61–74 (2010).

[6] Goobich, J. The real cost of content: It could be greater than you think (2021). https://www.forbes.com/councils/forbescommunicationscouncil/2021/08/17/the-real-cost-of-content-it-could-be-greater-than-you-think/. Accessed: 2025-01-07.

[7] Huang, Y. et al. Recent advances in artificial intelligence for video production system. *Enterprise Inf. Syst.* **17**, 2246188 (2023).

[8] Epstein, D., Jabri, A., Poole, B., Efros, A. & Holynski, A. Diffusion self-guidance for controllable image generation. *Adv. Neural Inf. Process. Syst.* **36**, 16222–16239 (2023).

[9] Colvert, A., Pothong, K. & Livingstone, S. Playful by design: Embedding children’s rights into the digital world. *ACM Games Res. Pract.* **2**, 1–10 (2024).

[10] Aslam, M., Evans, A. & Park, A. J. Natural language processing-driven text-to-animation framework. In *2024 IEEE 15th Annual Information Technology, Electronics and Mobile Communication Conference (IEMCON)*, 96–103 (IEEE, 2024).

[11] Zhou, R. et al. Multi-conditional generative adversarial network for text-to-video synthesis. *J. Comput. Aid. Des. Comput. Graph.* **34**, 1567–1579 (2022).

[12] Hannouni, S., El Filali, S. et al. Generative ai for education: A study of text-to-video generation for personalized learning. In *2025 International Conference on Circuit, Systems and Communication (ICCSC)*, 1–6 (IEEE, 2025).

[13] Song, X., Chen, J., Zhu, B. & Jiang, Y.-G. Text-driven video prediction. *ACM Trans. Multimed. Comput. Commun. Appl.* **20**, 1–15 (2024).

[14] Zhang, N. & Tang, H. Text-to-image synthesis: A decade survey. arXiv preprint arXiv:2411.16164 (2024).

[15] Goodfellow, I. et al. Generative adversarial networks. *Commun. ACM* **63**, 139–144 (2020).

[16] Zhu, M., Pan, P., Chen, W. & Yang, Y. Dm-gan: Dynamic memory generative adversarial networks for text-to-image synthesis. In *Proceedings of the 2019 IEEE/CVF Conference on Computer Vision and Pattern Recognition*, 5802–5810 (Long Beach, CA, USA, 2019 Oct 27-Nov 2).

[17] Xu, T. et al. Attngan: Fine-grained text to image generation with attentional generative adversarial networks. In *Proceedings of the 2018 IEEE Conference on Computer Vision and Pattern Recognition*, 1316–1324 (Salt Lake City, UT, USA, 2018 Jun 18-23).

[18] Zhang, H. et al. Stackgan: Text to photo-realistic image synthesis with stacked generative adversarial networks. In *Proceedings of the 2017 IEEE International Conference on Computer Vision*, 5907–5915 (Venice, Italy, 2017 Oct 22-29).

[19] Chen, H. et al. Pre-trained image processing transformer. In *Proceedings of the 2021 IEEE/CVF Conference on Computer Vision and Pattern Recognition*, 12299–12310 (Nashville, TN, USA, 2021 Jun 20-25).

[20] Ramesh, A. et al. Zero-shot text-to-image generation. In *International Conference on Machine Learning*, 8821–8831 (Pmlr, 2021 Jul 18-24).

[21] Radford, A. et al. Learning transferable visual models from natural language supervision. In *International Conference on Machine Learning*, 8748–8763 (PMLR, 2021 Jul 18-24).

[22] Cao, P., Zhou, F., Song, Q. & Yang, L. Controllable generation with text-to-image diffusion models: A survey. arXiv preprint arXiv:2403.04279 (2024). 10.1109/TPAMI.2025.3646548 41418008

[23] Ho, J., Jain, A. & Abbeel, P. Denoising diffusion probabilistic models. *Adv. Neural Inf. Process. Syst.* **33**, 6840–6851 (2020).

[24] Nichol, A. et al. Glide: Towards photorealistic image generation and editing with text-guided diffusion models. arXiv preprint arXiv:2112.10741 (2021).

[25] Saharia, C. et al. Photorealistic text-to-image diffusion models with deep language understanding. *Adv. Neural Inf. Process. Syst.* **35**, 36479–36494 (2022).

[26] Rombach, R., Blattmann, A., Lorenz, D., Esser, P. & Ommer, B. High-resolution image synthesis with latent diffusion models. In *Proceedings of the 2022 IEEE/CVF Conference on Computer Vision and Pattern Recognition*, 10684–10695 (New Orleans, LA, USA, 2022 Jun 18-24).

[27] Services, A. W. Unleashing stability AI’s most advanced text-to-image models for media, marketing, and advertising: Revolutionizing creative workflows (2024). https://aws.amazon.com/blogs/machine-learning/unleashing-stability-ais-most-advanced-text-to-image-models-for-media-marketing-and-advertising-revolutionizing-creative-workflows. Accessed: 2025-01-07.

[28] Deng, K. Creative applications of text-to-images: How artists and designers are using ai to generate visual content (2023). https://medium.com/@dengqs/creative-applications-of-text-to-images-how-artists-and-designers-are-using-ai-to-generate-visual-13ae9f84c2ab. Accessed: 2025-01-07.

[29] Imagine.art. Exploring the world of text-to-image ai: Applications and examples (2023). https://www.imagine.art/blogs/exploring-the-world-of-text-to-image-ai-applications-and-examples. Accessed: 2025-01-07.

[30] Chen, H. et al. Videocrafter2: Overcoming data limitations for high-quality video diffusion models. In *Proceedings of the 2024 IEEE/CVF Conference on Computer Vision and Pattern Recognition*, 7310–7320 (Seattle, WA, USA, 2024 June 16-22).

[31] Yang, Z. et al. Cogvideox: Text-to-video diffusion models with an expert transformer. arXiv preprint arXiv:2408.06072 (2024).

[32] Jin, Y. et al. Pyramidal flow matching for efficient video generative modeling. arXiv preprint arXiv:2410.05954 (2024).

[33] Wang, A. et al. Wan: Open and advanced large-scale video generative models. arXiv preprint arXiv:2503.20314 (2025).

[34] Zhang, L., Rao, A. & Agrawala, M. Adding conditional control to text-to-image diffusion models. In *Proceedings of the 2023 IEEE/CVF International Conference on Computer Vision*, 3836–3847 (Paris, France, 2023 Oct 1-6).

[35] Zhao, L., Peng, X., Tian, Y., Kapadia, M. & Metaxas, D. Learning to forecast and refine residual motion for image-to-video generation. In *Proceedings of the 2018 European Conference on Computer Vision (ECCV)*, 387–403 (Munich, Germany, 2018 Sep 8-14).

[36] Girdhar, R. et al. Emu video: Factorizing text-to-video generation by explicit image conditioning. arXiv preprint arXiv:2311.10709 (2023).

[37] Huang, K., Sun, K., Xie, E., Li, Z. & Liu, X. T2i-compbench: A comprehensive benchmark for open-world compositional text-to-image generation. *Adv. Neural Inf. Process. Syst.* **36**, 78723–78747 (2023).

[38] Orgad, H., Kawar, B. & Belinkov, Y. Editing implicit assumptions in text-to-image diffusion models. In *Proceedings of the 2023 IEEE/CVF International Conference on Computer Vision*, 7053–7061 (Paris, France, 2023 Oct 1-6).

[39] Chowdhery, A. et al. Palm: Scaling language modeling with pathways. *J. Mach. Learn. Res.* **24**, 1–113 (2023).

[40] Devlin, J. Bert: Pre-training of deep bidirectional transformers for language understanding. arXiv preprint arXiv:1810.04805 (2018).

[41] Dubey, A. et al. The llama 3 herd of models. arXiv preprint arXiv:2407.21783 (2024).

[42] Ho, J. et al. Cascaded diffusion models for high fidelity image generation. *J. Mach. Learn. Res.* **23**, 1–33 (2022).

[43] Luo, C. Understanding diffusion models: A unified perspective. arxiv 2022. arXiv preprint arXiv:2208.11970 (2022).

[44] Pernias, P., Rampas, D., Richter, M. L., Pal, C. J. & Aubreville, M. Würstchen: An efficient architecture for large-scale text-to-image diffusion models. arXiv preprint arXiv:2306.00637 (2023).

[45] Yang, Z. *et al.* Reco: Region-controlled text-to-image generation. In *Proceedings of the 2023 IEEE/CVF Conference on Computer Vision and Pattern Recognition*, 14246–14255 (Paris, France, 2023 Oct 1-6).

[46] Yang, Y. et al. Glyphcontrol: Glyph conditional control for visual text generation. *Adv. Neural Inf. Process. Syst.* **36**, 1–17 (2024).

[47] Schuhmann, C. et al. Laion-5b: An open large-scale dataset for training next generation image-text models. *Adv. Neural. Inf. Process. Syst.* **35**, 25278–25294 (2022).

[48] Lin, T.-Y. et al. Microsoft coco: Common objects in context. In *Computer Vision–ECCV 2014: 13th European Conference, Zurich, Switzerland, September 6-12, 2014, Proceedings, Part V 13*, 740–755 (Springer, 2014).

[49] Hessel, J., Holtzman, A., Forbes, M., Bras, R. L. & Choi, Y. Clipscore: A reference-free evaluation metric for image captioning. arXiv preprint arXiv:2104.08718 (2021).

[50] Wang, J., Chan, K. C. & Loy, C. C. Exploring clip for assessing the look and feel of images. In *Proceedings of the AAAI Conference on Artificial Intelligence*, vol. 37, 2555–2563 (Washington, DC, USA, 2023 Feb 7-14).

[51] Hu, Y. et al. Tifa: Accurate and interpretable text-to-image faithfulness evaluation with question answering. In *Proceedings of the 2023 IEEE/CVF International Conference on Computer Vision*, 20406–20417 (Paris, France, 2023 Oct 1-6).

[52] Huang, Z. et al. Vbench: Comprehensive benchmark suite for video generative models. In *Proceedings of the IEEE/CVF Conference on Computer Vision and Pattern Recognition*, 21807–21818 (2024).

[53] Mullan, J., Crawbuck, D. & Sastry, A. Hotshot-XL (2023).

[54] Wei, J. et al. Chain-of-thought prompting elicits reasoning in large language models. *Adv. Neural. Inf. Process. Syst.* **35**, 24824–24837 (2022).

[55] Ouyang, L. et al. Training language models to follow instructions with human feedback. *Adv. Neural. Inf. Process. Syst.* **35**, 27730–27744 (2022).

[56] Okamoto, H. & Tachizaki, H. A study of image editing ai awareness in children: Can ai that lets still images speak capture the interest of young children?. *J. Educ. Technol. Dev. Exchange (JETDE)* **18**, 117–126 (2025).

[57] Tang, X., Zainal, S. R. B. M. & Li, Q. Multimedia use and its impact on the effectiveness of educators: a technology acceptance model perspective. *Hum. Soc. Sci. Commun.* **10**, 1–11 (2023).

[58] Zarei, A. A. & Salimi, A. The comparative effects of song, picture and the keyword method on l2 vocabulary recognition and production. *Appl. Res. English Lang.* **1**, 43–56 (2012).

[59] Lee, S. H. & Aspiranti, K. B. Using multimodal educational apps to increase the vocabulary of children with and without reading difficulties. *Int. J. Child-Comput. Interact.* **36**, 100579 (2023).

[60] Undheim, M. Children and teachers engaging together with digital technology in early childhood education and care institutions: A literature review. *Eur. Early Child. Educ. Res. J.* **30**, 472–489 (2022).

[61] Xing, Z. et al. A survey on video diffusion models. *ACM Comput. Surv.* **57**, 1–42 (2024).

[62] Chandrabanshi, V. & Domnic, S. Leveraging 3d-cnn and graph neural network with attention mechanism for visual speech recognition: V. chandrabanshi, s. domnic. *Signal Image Video Process.* **19**, 844 (2025).

[63] Chandrabanshi, V. & Domnic, S. A novel framework using 3d-cnn and bilstm model with dynamic learning rate scheduler for visual speech recognition. *SIViP* **18**, 5433–5448. 10.1007/s11760-024-03245-7 (2024).

[64] Sung-Bin, K., Senocak, A., Ha, H., Owens, A. & Oh, T.-H. Sound to visual scene generation by audio-to-visual latent alignment. In *Proceedings of the IEEE/CVF Conference on Computer Vision and Pattern Recognition*, 6430–6440 (2023).

[65] Xiang, J., Zhu, X. & Cambria, E. Integrating audio–visual text generation with contrastive learning for enhanced multimodal emotion analysis. *Inf. Fusion* 103809 (2025).

[66] Tam, T. Y. C. et al. A framework for human evaluation of large language models in healthcare derived from literature review. *NPJ Digit. Med.* **7**, 258 (2024). 39333376 10.1038/s41746-024-01258-7 PMC11437138

[67] Bender, E. M., Gebru, T., McMillan-Major, A. & Shmitchell, S. On the dangers of stochastic parrots: Can language models be too big? In *Proceedings of the 2021 ACM conference on fairness, accountability, and transparency*, 610–623 (2021).

[68] Vilaca, L., Yu, Y. & Viana, P. A survey of recent advances and challenges in deep audio-visual correlation learning. *ACM Comput. Surv.* **57**, 1–46 (2025).

