# Supplementary material for: Automatic background animation generation aligned with LLM-generated lyrics for children’s songs
Source: Sci Rep. 2025 Dec 27;16:591. doi: 10.1038/s41598-025-30139-6 (PMC12775371; doi:10.1038/s41598-025-30139-6)
Supplement: Supplementary file 1 — Supplementary Information. [file 41598_2025_30139_MOESM1_ESM.pdf]

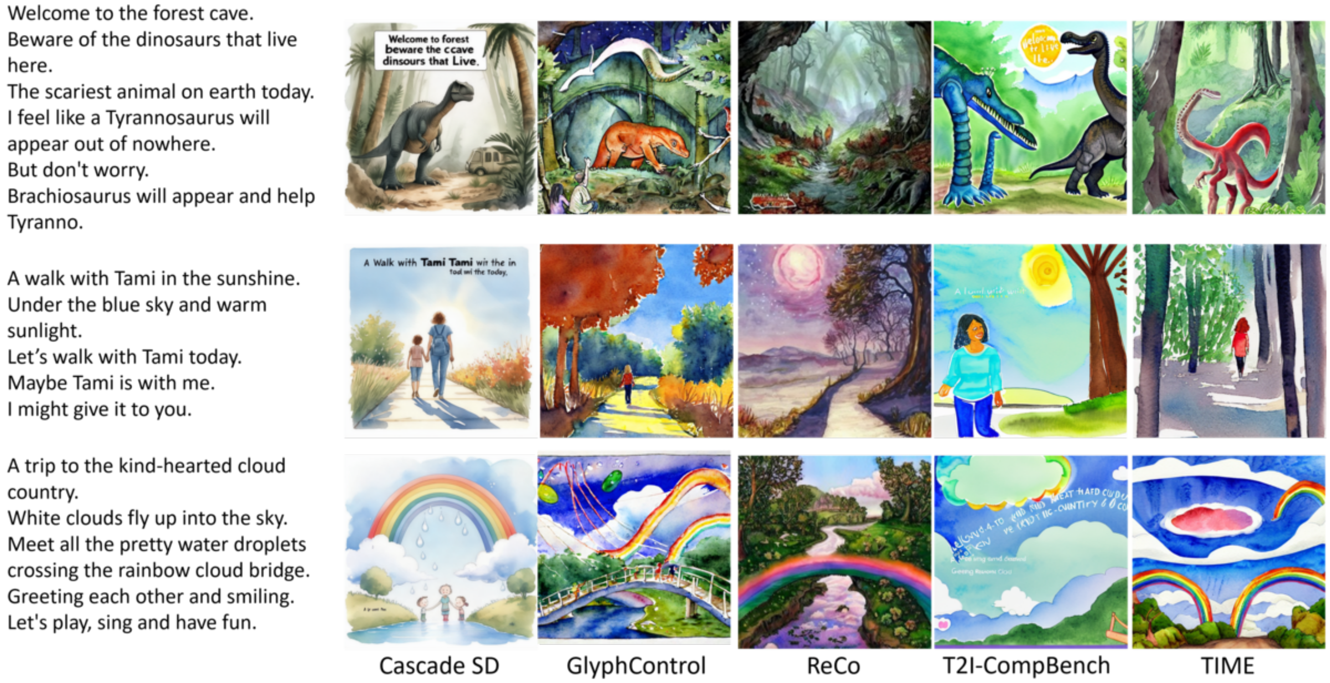

Supplementary Figure S1. Illustrate a watercolor scene inspired by the following lyrics: < *lyrics* >. All images shown are generated by the five models.

Supplementary Table S1. Performance of different CascadeSD configurations. Inference times were measured in seconds per image.

Stage B	Stage C	CLIP score	CLIP-IQA	TIFA	Inference time
small	small	0.370±0.025	0.717±0.085	**0.746±0.199**	**4.69±0.59**
small	large	**0.371±0.037**	0.701±0.082	0.718±0.249	6.16±0.59
large	small	0.370±0.027	0.762±0.104	0.719±0.209	8.69±0.74
large	large	0.359±0.029	**0.792±0.102**	0.753±0.199	10.38±0.78

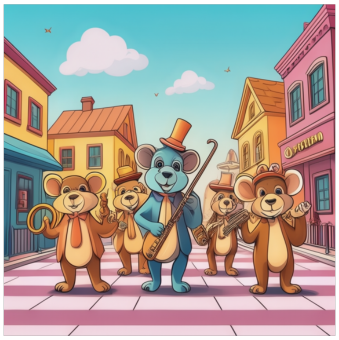

A lively parade of cartoon animals marching through a candy-themed town, each playing a musical instrument, including a giraffe on a trombone and a monkey with cymbals, done in a 3D digital style with bright, vibrant colors.

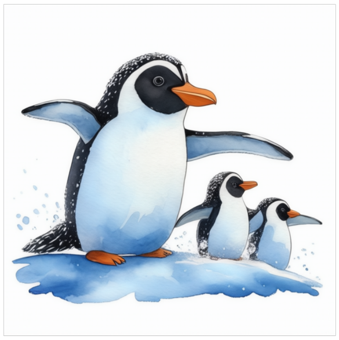

A penguin sliding down an icy hill while singing, with other penguins clapping and cheering, painted in a child-friendly watercolor style.

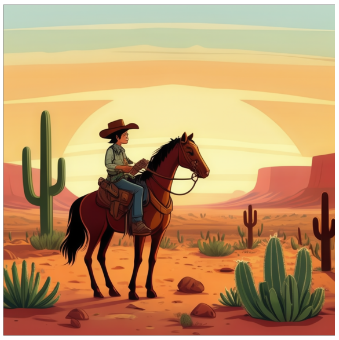

A cowboy boy and girl playing harmonicas on horseback, riding through a desert with cacti and a sunset, illustrated in a cartoon Western style.

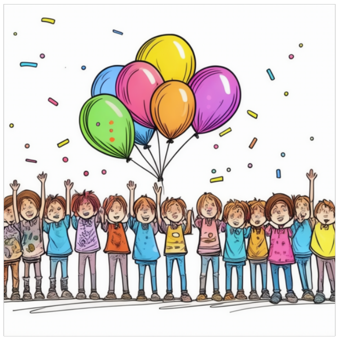

A group of children singing together on a school stage with colorful balloons and confetti falling, drawn in vibrant marker and ink.

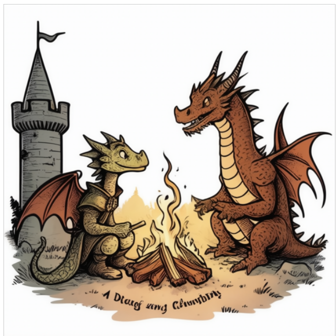

A dragon and a knight sitting around a campfire, singing songs together, with a castle silhouetted in the distance, in a storybook style with bold ink outlines.

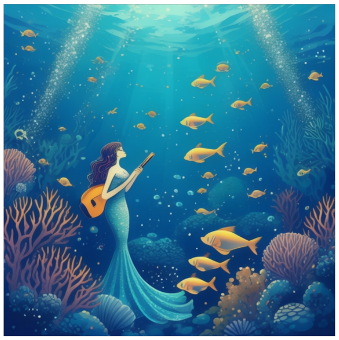

An underwater scene with a mermaid choir performing for a school of fish, surrounded by coral reefs and bubbles, drawn in a glittery digital art style.

Supplementary Figure S2. Examples of difficult prompt scenarios visualization. All images shown are generated by CascadeSD. The output image is generated by CascadeSD.

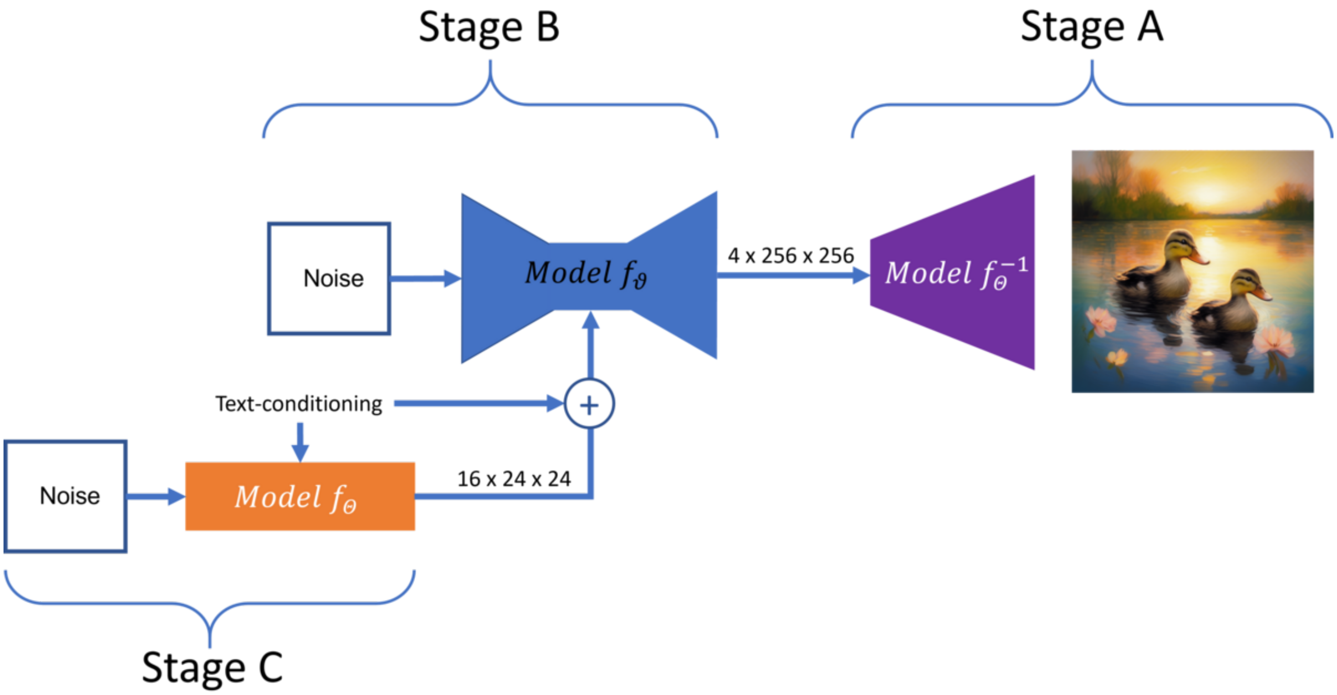

Supplementary Figure S3. Architectural overview of CascadeSD model. The output image is generated by CascadeSD.

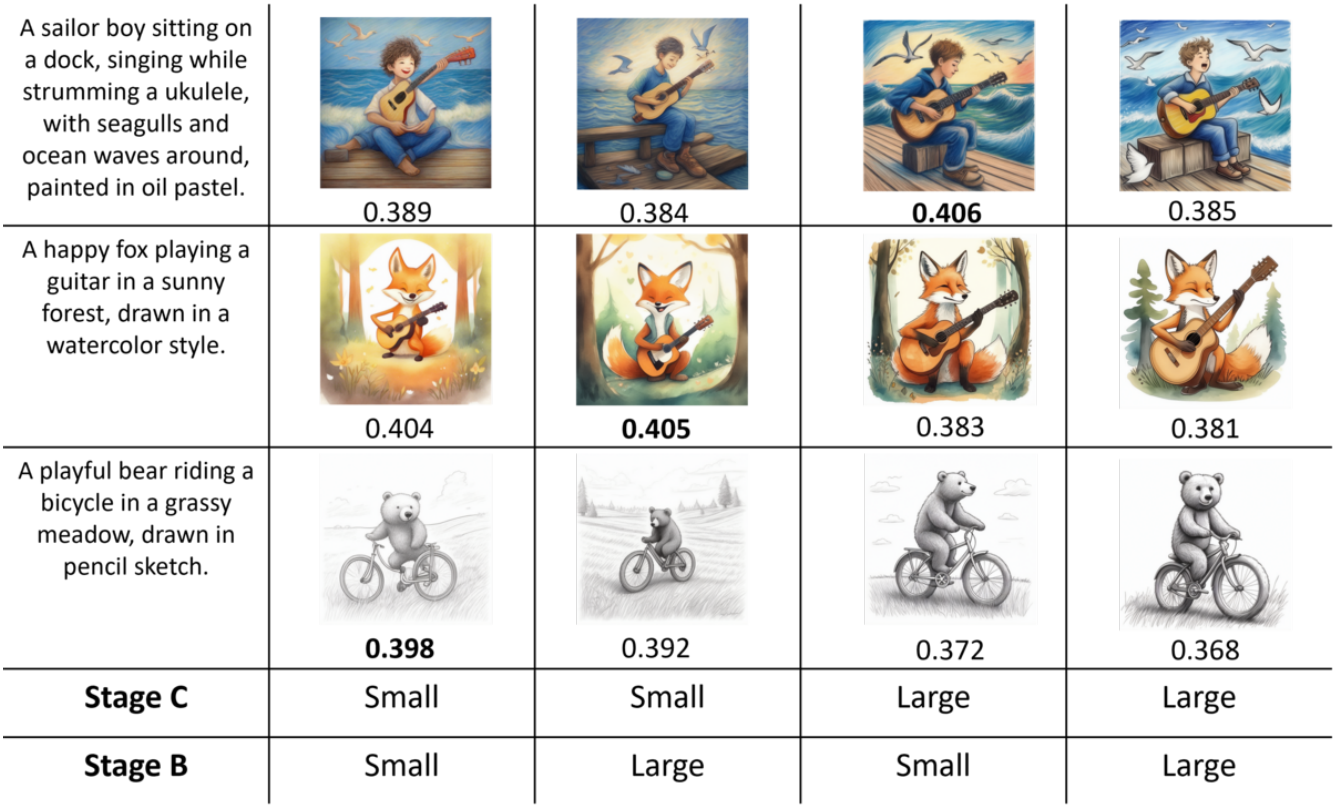

Supplementary Figure S4. Images generated by different CascadeSD configurations. All images are generated by CascadeSD.

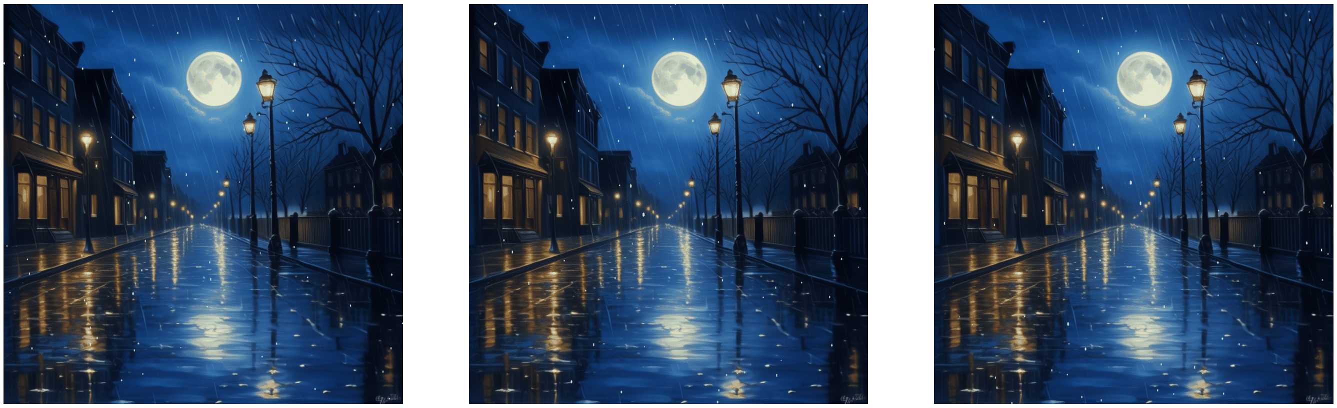

Start Middle End

(a) Animation of a "calm rainy street scene at night with reflections on wet pavement".

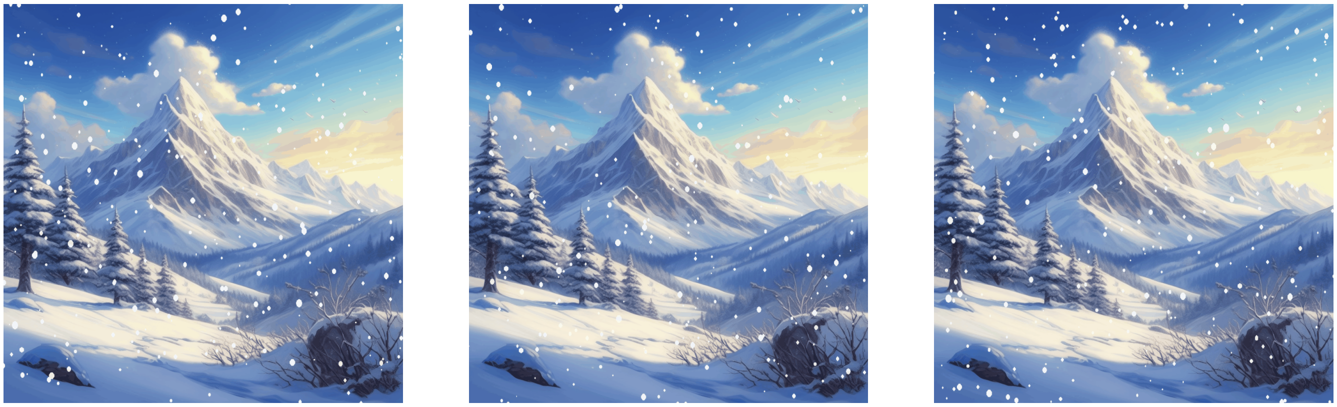

Start Middle End

(b) Animation of a "peaceful mountain landscape covered in snow".

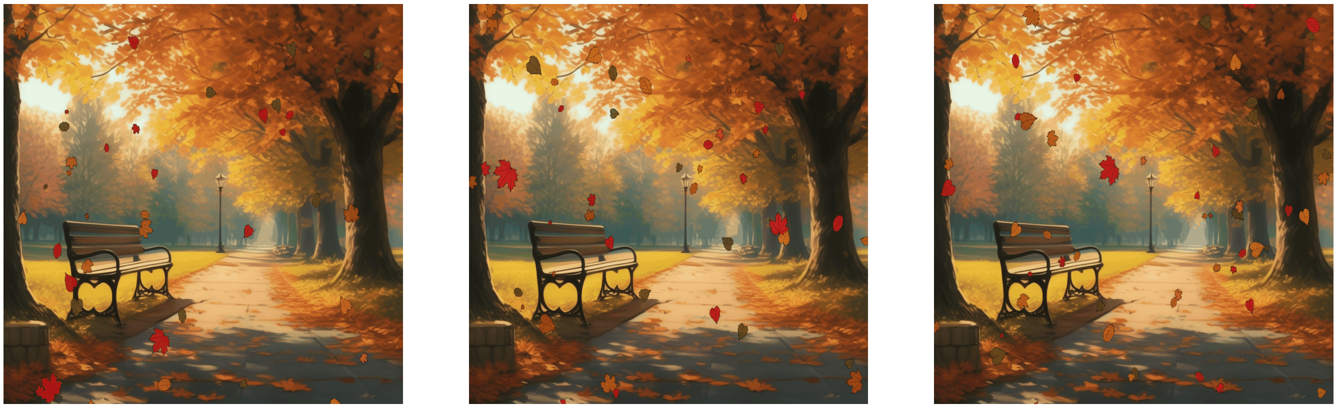

Start Middle End

(c) Animation of a "quiet park bench under falling autumn leaves".

Supplementary Figure S5. Examples of generated animations by our pipeline. Each row shows the start, middle, and end frames of a generated animation. Each frame is generated by CascadeSD.
